# Cardiometabolic Candidate Endotypes in Psoriatic Disease: Integration of Clinical, Metabolic, and Immunogenetic Data Across Psoriasis and Psoriatic Arthritis

**DOI:** 10.3390/life16010002

**Published:** 2025-12-19

**Authors:** Rubén Queiro, Paula Alvarez, Ignacio Braña, Marta Loredo, Estefanía Pardo, Stefanie Burger, Norma Callejas, Sara Alonso, Mercedes Alperi

**Affiliations:** 1 Rheumatology Division, Central University Hospital of Asturias, 33011 Oviedo, Spain; paulalvarez992@gmail.com (P.A.); i.brana.abascal@hotmail.es (I.B.); mloredomart@gmail.com (M.L.); estefaniapardoc@gmail.com (E.P.); stefanie.nam@gmail.com (S.B.); normaale9222@gmail.com (N.C.); saraalonsocastro@hotmail.com (S.A.); mercedes_alperi@hotmail.com (M.A.); 2 Department of Medicine, Oviedo University School of Medicine, 33006 Oviedo, Spain; 3 Translational Immunology Division, Biohealth Research Institute of the Principality of Asturias (ISPA), 33011 Oviedo, Spain

**Keywords:** psoriasis, psoriatic arthritis, cardiovascular, HLA-Cw6, endotypes, unsupervised clustering

## Abstract

Background/objectives: Psoriatic disease (PsD) encompasses psoriasis (PsO) and psoriatic arthritis (PsA) and is associated with heterogeneous cardiometabolic risk. Integrating immunogenetic markers such as HLA-Cw6 into data-driven analyses may refine phenotyping and uncover clinically meaningful endotypes. We aimed to identify cardiometabolic phenotypes across PsD, integrating HLA-Cw6 and exploring disease-specific heterogeneity and predictors of high-risk profiles. Methods: In a cross-sectional study of 572 PsD patients (401 PsO, 171 PsA), eight demographic and clinical variables, including HLA-Cw6, were entered into k-means clustering (k = 4). Cardiometabolic risk factors were profiled post hoc. Cluster validity was assessed by Gaussian Mixture Models and principal component analysis (PCA). Stratified analyses (k = 3) were conducted separately for PsO and PsA. Predictors of the high-risk phenotype were examined using bootstrap-resampled logistic regression. Results: Four cardiometabolic phenotypes were identified, ranging from younger patients with active PsO and low cardiometabolic burden to a small, high-risk subgroup (~6%) combining older age, universal cardiovascular disease, and a clustering of hypertension, diabetes, and dyslipidemia. Disease-stratified analyses showed that high-risk phenotypes were present in both PsO and PsA. In stratified analyses, HLA-Cw6 showed opposite associations—enriched in high-risk PsO (OR 2.0, 95% CI 1.3–3.1) but depleted in high-risk PsA (OR 0.24, 95% CI 0.11–0.52). Conclusions: Incorporating HLA-Cw6 into clustering identified reproducible cardiometabolic phenotypes with distinct genetic signatures. The inverse HLA-Cw6 risk patterns in PsO and PsA suggest disease-specific patterns that may have differing cardiometabolic implications, which should be tested in longitudinal studies.

## 1. Introduction

Psoriatic disease (PsD) encompasses a heterogeneous group of inflammatory disorders, including psoriasis (PsO) and psoriatic arthritis (PsA), characterized by variable involvement of the skin, nails, peripheral joints, axial skeleton, and entheses. Beyond musculoskeletal and cutaneous domains, PsD is associated with a substantially increased burden of cardiometabolic comorbidity, including obesity, hypertension, diabetes mellitus, dyslipidemia, hyperuricemia, non-alcoholic fatty liver disease, and established cardiovascular disease (CVD). The excess cardiovascular risk in PsD is driven by a combination of traditional risk factors, systemic inflammation, and potential adverse effects of certain therapies. Psoriasis and PsA are now recognized as systemic immune-mediated inflammatory diseases in which low-grade, chronic inflammation involving the IL-23/IL-17 axis, TNF, and adipokine dysregulation not only drives cutaneous and musculoskeletal disease but also contributes to endothelial dysfunction, insulin resistance, and accelerated atherosclerosis. Consequently, patients with PsD bear a disproportionate burden of hypertension, dyslipidemia, diabetes, and major adverse cardiovascular events. These comorbidities contribute to reduced life expectancy, poorer quality of life, and higher healthcare costs [1,2,3]. In addition, systemic therapies used in PsD may modulate cardiometabolic risk. TNF and IL-17/IL-23 pathway inhibitors can improve systemic inflammation and have been associated with favorable changes in some metabolic parameters and vascular surrogates in observational studies. However, evidence for a consistent reduction in hard cardiovascular outcomes remains limited and heterogeneous across drug classes and study designs [1,2]. Against this background, identifying clinical–genetic phenotypes with distinct cardiometabolic profiles may help to tailor both systemic anti-inflammatory therapy and cardiovascular prevention strategies.

Although numerous epidemiological studies have established the association between PsD and increased cardiometabolic risk, most research has treated these comorbidities as isolated variables rather than as components of integrated phenotypes. This approach may overlook clinically relevant subgroups in which specific constellations of disease features and comorbidities cluster together, leading to different prognoses and therapeutic needs [1,2,3]. Identifying such “cardiometabolic endotypes” could facilitate more personalized risk stratification and management strategies, allowing clinicians to target preventive and therapeutic interventions more effectively [4].

The concept of endotyping—subdividing a heterogeneous disease into biologically or clinically distinct subgroups—has been successfully applied in other chronic inflammatory disorders such as asthma and inflammatory bowel disease [5]. In rheumatology, cluster analysis and latent class analysis (LCA) have been increasingly used to uncover phenotypes in diseases like systemic lupus erythematosus and axial spondyloarthritis [6,7]. However, comparable approaches in PsD focusing on cardiometabolic clustering remain scarce, and studies integrating both PsO and PsA populations in the same analytic framework are virtually absent.

Given the diversity of PsD presentations, it is plausible that the cardiometabolic burden varies not only in magnitude but also in qualitative composition across subgroups. For example, some patients may present with high skin activity and minimal joint disease but accumulate significant metabolic risk factors, whereas others may display severe musculoskeletal involvement with a relatively benign metabolic profile. Understanding these patterns could improve clinical monitoring, optimize therapeutic choices, and inform cardiovascular prevention programs.

In this study, we applied unsupervised clustering methods to a well-characterized cohort of patients with PsD, including both PsO-only and PsA subgroups, to identify and characterize distinct cardiometabolic phenotypes. We focused on core demographic and disease-related variables (age, sex, disease duration, arthritis status, HLA-Cw6, PASI, systemic treatment, and cardiovascular disease history) to derive the clusters and then profiled each subgroup according to the prevalence of major cardiovascular risk factors (hypertension, diabetes, dyslipidemia, obesity, smoking, and liver disease). We further validated our findings using a model-based LCA approach, explored disease-stratified clustering, and evaluated predictors of the “high-risk” phenotype. Here we use the term ‘candidate endotypes’ in an exploratory sense, acknowledging that full endotype validation would require multi-omics and mechanistic studies. With this in mind, we aimed to identify candidate cardiometabolic phenotypes/endotypes across PsD and to discuss their potential implications for individualized patient care and future research.

## 2. Materials and Methods

### 2.1. Study Design and Population

We performed a cross-sectional observational study including adult patients with PsD, defined as either PsO without arthritis or PsA fulfilling the CASPAR criteria [8], who attended a specialized dermatology-rheumatology clinic. The study included consecutive adult patients attending the dermatology–rheumatology joint clinic at the Hospital Universitario Central de Asturias. Given the exploratory nature of the analysis, the sample size corresponded to all eligible patients with complete data during the recruitment period. All patients had complete demographic and clinical data available for the variables of interest and provided informed consent for the use of anonymized data for research purposes. The study was conducted in accordance with the Declaration of Helsinki and approved by the Ethics Committee of the Hospital Universitario Central de Asturias (approval number CEImPA-214/2023).

### 2.2. Variables

For cluster derivation, we selected eight core variables based on their clinical relevance and potential relationship to cardiometabolic status: age (years), disease duration, sex, arthritis status, HLA-Cw6 status, Psoriasis Area and Severity Index (PASI), systemic therapy use, and cardiovascular disease history. The following cardiovascular risk factors were excluded from the clustering procedure and used exclusively for profiling the resulting clusters: high blood pressure (HBP), diabetes mellitus type 1 or type 2 (DM), dyslipidemia, obesity (BMI ≥ 30 kg/m^2^), current smoking, and liver disease (non-alcoholic fatty liver disease). Dyslipidemia was recorded when total cholesterol, LDL cholesterol, HDL cholesterol, and/or triglyceride levels met standard diagnostic thresholds or when patients were receiving lipid-lowering therapy, according to current clinical guidelines. Non-alcoholic fatty liver disease (NAFLD) was recorded when explicitly diagnosed in the medical record by hepatology or gastroenterology based on routine abdominal imaging (primarily ultrasound) and laboratory assessment, in the absence of alternative causes of chronic liver disease.

### 2.3. Data Preprocessing

Binary variables were encoded as 0/1. Continuous variables (age, disease duration, PASI) were retained as numeric values. Missing values were imputed using the median for continuous and binary variables alike. All clustering variables were standardized to z-scores prior to analysis to ensure equal contribution to the distance metrics.

### 2.4. Clustering Procedures

We applied k-means clustering for k values from 3 to 6, each with 50 random initializations. Cluster validity was assessed using the silhouette coefficient, the Calinski–Harabasz index, and the within-cluster sum of squares (inertia). The number of clusters was chosen based on optimal validity indices and clinical interpretability. To avoid overweighting highly correlated cardiometabolic variables in the distance metric, we restricted the clustering model to eight core demographic, clinical, and immunogenetic variables (age, disease duration, sex, PsA status, HLA-Cw6, PASI, systemic therapy, and prior CVD). Classical cardiovascular risk factors (hypertension, diabetes, dyslipidemia, obesity, smoking, and NAFLD) were reserved for post hoc profiling of the resulting clusters.

### 2.5. Validation and Additional Analyses

To validate the primary k-means solution (model-based internal validation/sensitivity analysis), we conducted a latent class analysis (LCA)-like approach using Gaussian mixture models (GMM) with diagonal covariance matrices. The best-fitting model was selected according to the Bayesian Information Criterion (BIC). Agreement between k-means and GMM classifications was quantified using the adjusted Rand index (aRi). We also performed disease-stratified clustering by repeating the k-means procedure (k = 3) separately in PsO-only and PsA subgroups to identify within-disease phenotypes. Correlation analysis of clustering variables revealed moderate correlations only (all |r| ≤ 0.43), supporting the suitability of these variables for k-means clustering (Figure S1). Finally, we conducted a binary logistic regression (highest CV risk cluster vs. all other clusters) to identify independent predictors of belonging to the “high-risk” phenotype. Continuous predictors were standardized; odds ratios (OR) and 95% confidence intervals (CI) were estimated using bootstrap resampling (B = 500). A sensitivity analysis was conducted by re-running k-means clustering with additional anthropometric variables (BMI, waist circumference).

### 2.6. Cluster Profiling and Visualization

Clusters were described using medians and interquartile ranges [IQR] for continuous variables and percentages for categorical variables. Cardiometabolic risk burden was summarized as the median [IQR] number of risk factors (range 0–6) per patient. We generated heatmaps of normalized (0–1) mean values for each variable across clusters, both for the entire cohort and separately for PsO-only and PsA subgroups, to illustrate relative patterns of clinical and metabolic features. To visualize and summarize the overall structure of the dataset, a principal component analysis (PCA) was performed using all clinical, genetic, and cardiometabolic variables. The first two components were used to display the relative position of the four clusters. Variables were standardized before PCA, and loadings were inspected to identify the main contributors to each component.

### 2.7. Software

All analyses were performed in Python 3.10 using pandas, scikit-learn, numpy, and matplotlib libraries.

## 3. Results

### 3.1. Summary of Study Population

A total of 572 patients with PsD were included, of whom 401 (70.1%) had PsO without arthritis and 171 (29.9%) had PsA. The mean age at assessment was 46.7 (SD 14.5) years, with a median disease duration of 17.0 years [8.0–30.0]. Females represented 46% of the cohort. The average BMI was 27.6 (SD 5.02), while the average waist circumference was 98 cm (min: 61, max: 138). Just over a third (34.3%) of the patients were smokers, while the median alcohol consumption according to standard drink units (SDU) was 0 (min: 0, max: 30). Fatty liver disease was ruled out in 405 (70.8%), confirmed in 129 (22.6%) and no information was available for 38 (6.6%). A total of 20% of the patients had hypertension, and another 20% had dyslipidemia. In total, 33 patients had adverse coronary events (5.8%), 22 patients were type I diabetics (3.8%), while 45 (7.9%) were type 2. A total of 241 patients expressed the HLA-Cw6 allele (42.1%). The remaining diseases features, as well as sex-based differences, are shown in Table 1.

### 3.2. Unsupervised Clustering (k-Means)

The optimal k-means solution was obtained with four clusters (k = 4). Silhouette coefficients favored the 4-cluster solution (0.22) over k = 3, 5, or 6. Main characteristics of the 4 cluster were as follows:

- Cluster 1 (C1, n = 217; 37.9%)—“Active PsO, low CV risk”. Psoriasis-only (0% arthritis) with high PASI (median 12.0 [9.0–20.0]), almost universal systemic therapy (99.5%), relatively young (median 45 years), and low prevalence of cardiometabolic factors (HBP 13.8%, DM 7.4%, dyslipidemia 13.4%).
- Cluster 2 (C2, n = 144; 25.2%)—“Inflammatory PsA, moderate CV risk”. Exclusively PsA (100%), median PASI 12.0 [8.0–20.0], high systemic therapy use (93.8%), older than C1 (median 49 years), and intermediate cardiometabolic profile (HBP 20.8%, DM 13.2%, dyslipidemia 25.0%).
- Cluster 3 (C3, n = 178; 31.1%)—“Mild PsO, high smoking prevalence”. Predominantly PsO (92.7%), lowest PASI (4.0 [3.0–6.0]), no systemic therapy, youngest group (median 43.5 years), and highest smoking prevalence (41.0%), with otherwise low–moderate cardiometabolic burden.
- Cluster 4 (C4, n = 33; 5.8%)—“High cardiometabolic risk”. Mixed PsO/PsA (42.4% arthritis), older age (median 66.0 years), longest disease duration (30 years), universal cardiovascular disease (100%), and highest prevalence of all risk factors (HBP 75.8%, DM 45.5%, dyslipidemia 69.7%). Median number of risk factors was 3 [2,3,4].
- Patients in the high-risk phenotype (C4) showed the longest median disease duration (30 years), exceeding that of C1–C3. Although cross-sectional, this finding suggests that cumulative disease exposure may contribute to the aggregation of cardiometabolic burden

**Figure 1:**
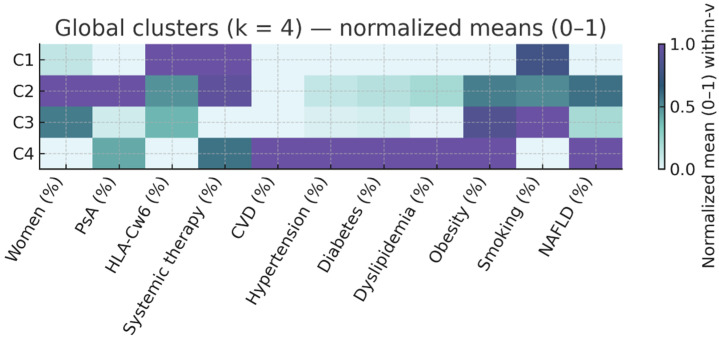
Overall Cluster Profile Heatmap. Heatmap displays normalized means (0–1) across clusters for clinical and cardiometabolic variables. Darker color intensity indicates higher relative values within-variable. Variables include age, disease duration, sex, arthritis, Cw6, PASI, systemic therapy, cardiovascular disease, and cardiovascular risk factors (HTN, diabetes [type 1/2], dyslipidemia, obesity BMI ≥ 30, smoking, fatty liver disease). C1: Active PsO, low CV risk; C2: Inflammatory PsA, moderate CV risk; C3: Mild PsO with high smoking prevalence; C4: High cardiometabolic risk.

The resulting phenotypes are summarized in Table 2 and illustrated in Figure 1 (normalized heatmap of clinical and metabolic variables).

Bootstrap resampling (B = 2000) of the high-risk cluster yielded narrow confidence intervals for the prevalence of hypertension, diabetes, dyslipidemia, NAFLD, smoking, and BMI, supporting the internal stability of this subgroup despite its modest size (Table S3). Sensitivity clustering incorporating BMI and waist circumference reproduced an identical high-risk cluster (100% membership overlap), confirming robustness of this subgroup to model specification (Figure S2).

### 3.3. Validation with Gaussian Mixture Models (LCA-like)

Model-based clustering using GMM identified a best-fitting solution with k = 4 by BIC, closely paralleling the k-means structure. Agreement between classifications was high (aRi 0.83). The GMM-derived classes reproduced the same spectrum of phenotypes, confirming the robustness of the findings.

### 3.4. Disease-Stratified Clustering

**Figure 2:**
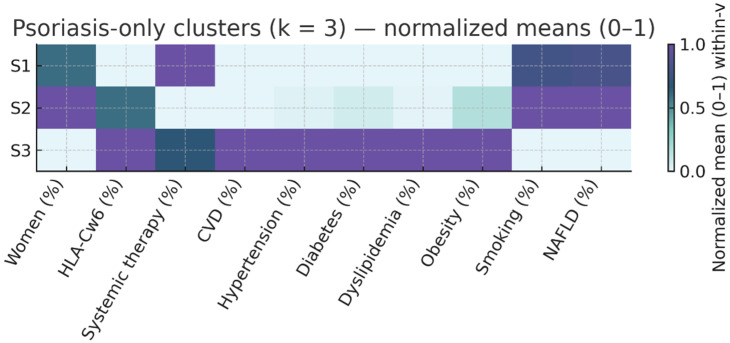
Psoriasis-only Cluster Profile Heatmap (k = 3). Same normalization as Figure 1, limited to patients without arthritis. Clusters S1–S3 highlight within-psoriasis phenotypic variability, including activity (PASI), treatment, and risk-factor load. S1: Active PsO, low CV risk; S2: Mild PsO, high smoking prevalence; S3: High cardiometabolic risk.

**Figure 3:**
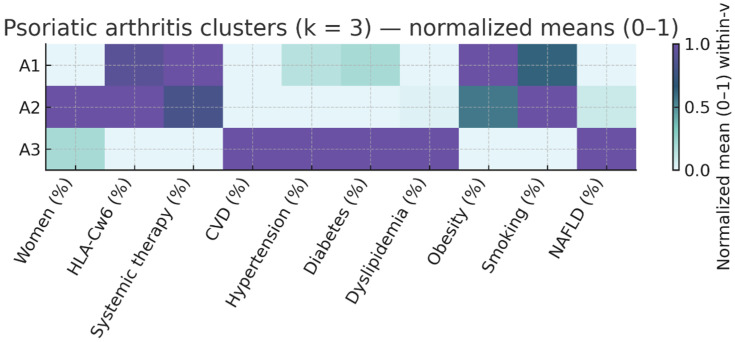
Psoriatic Arthritis Cluster Profile Heatmap (k = 3). Same normalization as Figure 1, restricted to patients with arthritis. Clusters S1–S3 capture within-PsA gradients in age/duration, PASI, treatment exposure, and cardiometabolic burden. A1: Inflammatory PsA, moderate CV profile; A2: Younger PsA with low CV burden; A3: Older PsA with high CV risk.

We repeated the k-means procedure with k = 3 separately in patients with PsO only and those with PsA to explore within-disease heterogeneity. In PsO-only patients, high cardiometabolic burden was concentrated in the S3 phenotype, which combined older age, established CVD, and multiple risk factors. In PsA, a distinct S3 phenotype also captured older patients with universal CVD and a heavy cardiometabolic load. The results are summarized in Table 3 and Table 4 and illustrated in Figure 2 and Figure 3.

In PsO three distinct phenotypes emerged:

- S1 (n = 217; 54.1% of PsO)—Active PsO, low CV risk. Median PASI 12.0 [9.0–20.0], almost universal systemic therapy (99.5%), median age 45 years, and low prevalence of hypertension (13.8%), diabetes (7.4%), and dyslipidemia (13.4%). Median number of CV risk factors: 1 [0–2]. HLA-Cw6 prevalence 34.6%.
- S2 (n = 165; 41.1% of PsO)—Mild PsO, high smoking prevalence. Lowest PASI (4.0 [3.0–6.0]), no systemic therapy, median age 44 years, highest smoking prevalence (40.6%), with otherwise modest metabolic risk (HBP 15.2%, DM 9.7%, dyslipidemia 13.9%). Median CV risk factors: 1 [1,2]. HLA-Cw6 prevalence 48.5%.
- S3 (n = 19; 4.7% of PsO)—PsO with elevated metabolic risk. Median PASI 8.0 [3.2–13.5], systemic therapy in 68.4%, older age (66 years), and disproportionately high metabolic burden (HBP 68.4%, DM 42.1%, dyslipidemia 68.4%, obesity 47.4%). Median CV risk factors: 2 [2,3,4]. HL A-Cw6 prevalence 57.9%.

In PsA three phenotypes were also identified:

- S1 (n = 93; 54.4% of PsA)—Inflammatory PsA, moderate CV profile. Median PASI 12.0 [7.4–20.0], systemic therapy in 89.2%, median age 52 years, and moderate cardiometabolic burden (HBP 25.8%, DM 16.1%, dyslipidemia 23.7%). Median CV risk factors: 1 [1,2]. HLA-Cw6 prevalence 46.2%
- S2 (n = 64; 37.4% of PsA)—Younger PsA with low CV burden. Median PASI 12.0 [6.1–16.0], systemic therapy in 81.2%, median age 47 years, low prevalence of HBP (15.6%), DM (7.8%), and obesity (21.9%). Median CV risk factors: 1 [0–2]. HLA.Cw6 prevalence 48.4%.
- S3 (n = 14; 8.2% of PsA)—Older PsA with high CV risk. Median PASI 4.0 [3.0–15.0], systemic therapy in 42.9%, median age 67 years, universal cardiovascular disease (100%), and highest metabolic burden (HBP 85.7%, DM 50.0%, dyslipidemia 71.4%). Median CV risk factors: 3 [2–3.8]. HLA-Cw6 prevalence 21.4%.

Leave-one-out resampling within the PsA subgroup showed that 12 of 14 PsA-S3 patients (86%) were consistently reassigned to the same high-risk phenotype, supporting its internal stability.

When the analysis was restricted to patients with PsO, the prevalence of HLA-Cw6 positivity increased progressively across clusters, reaching its highest value in the high-risk phenotype (C3: 57.9%) compared to the combined low/moderate-risk phenotypes (C1–C2: 40.6%). This difference was statistically significant (OR = 2.00, 95% CI 1.29–3.09, *p* = 0.0026). In contrast, in PsA, the high-risk phenotype (C3) exhibited a markedly lower prevalence of HLA-Cw6 positivity (21.4%) than the lower-risk phenotypes (C1–C2: 47.1%), yielding an inverse and statistically robust association (OR = 0.24, 95% CI 0.11–0.52, *p* = 0.00029).

**Figure 4:**
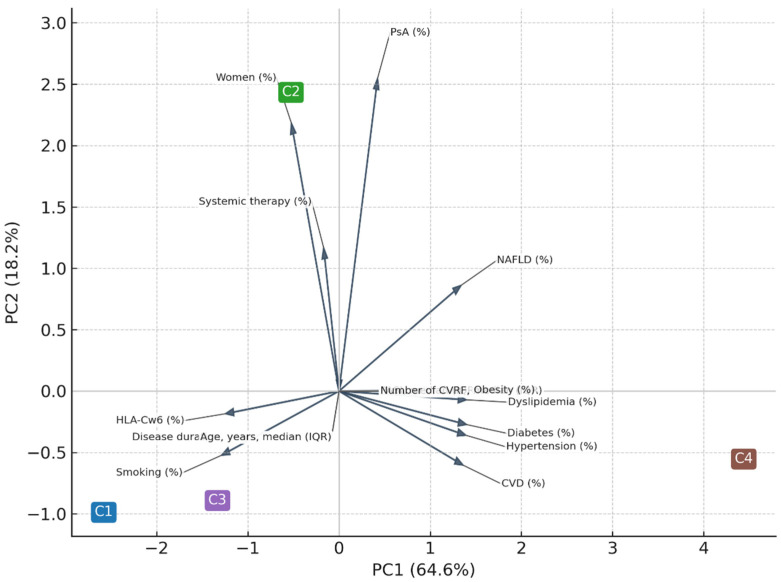
Principal component analysis (PCA) performed on cluster centroids. Principal component analysis (PCA) performed on cluster centroids, illustrating the relative positioning of the four clusters in the multidimensional space. Percentages of variance refer to between-cluster variance (PC1 = 64.6%, PC2 = 18.2%). PC1 mainly reflected the cardiometabolic axis (age, hypertension, diabetes, dyslipidemia, cardiovascular disease), whereas PC2 captured the inflammatory and demographic dimension (psoriatic arthritis, female sex, systemic therapy). Arrows indicate variable loadings, and their directions correspond to the axes of maximal correlation.

Principal component analysis (PCA) performed on individual-level data accounted for 69% of total variance (PC1 = 47%, PC2 = 22%). For graphical purposes, the PCA displayed in Figure 4 was computed using the cluster centroids, which explained 83% of between-cluster variance (PC1 = 64.6%, PC2 = 18.2%). Both approaches confirmed a dominant cardiometabolic axis (PC1) and a secondary inflammatory/immunogenetic axis (PC2).

The contribution of each variable to PCA is depicted in Table 5.

An age-adjusted PCA, derived from residualizing all variables against age, reproduced the same cardinal axes (cardiometabolic and inflammatory/immunogenetic). This indicates that the observed clustering structure is not driven solely by chronological age (Figure S3).

An HLA-Cw6 × disease-type interaction model confirmed opposite associations of HLA-Cw6 with the high-risk phenotype in PsA (interaction β = –2.34, *p* = 0.042), supporting disease-specific immunogenetic modulation of cardiometabolic risk (Table S4).

### 3.5. Predictors of High-Risk Phenotype

Three multivariable logistic regression models were built to identify factors associated with belonging to the high-risk cluster (C4). In the full model including all candidate variables (age, sex, PsA, PASI, HLA-Cw6, systemic therapy, hypertension, diabetes, dyslipidemia, and obesity), only age and cardiometabolic comorbidities—particularly hypertension and dyslipidemia—remained significantly associated with C4 membership. In a clinically adjusted model restricted to variables with *p* < 0.10 in univariate analysis, the same pattern was observed, confirming the dominant contribution of age and traditional metabolic risk factors. Finally, a backward stepwise model yielded a parsimonious configuration including age, hypertension, and dyslipidemia, which retained comparable explanatory power and discrimination (AUC ≈ 0.80). Collectively, these results indicate that the high-risk phenotype is largely driven by older age and cumulative cardiometabolic burden, rather than by psoriatic domain or genetic background (HLA-Cw6). Confidence intervals were calculated using bootstrap resampling (B = 500) to ensure robustness of the estimates. Full regression models and tests for collinearity diagnostics (VIF) are shown in Supplementary Tables S5 and S6.

## 4. Discussion

In this cross-sectional study of 572 patients with PsD, we identified four distinct cardiometabolic phenotypes using unsupervised k-means clustering applied to demographic, clinical, and immunogenetic data, including HLA-Cw6 status. These phenotypes differed markedly in age, disease duration, systemic therapy use, and cardiometabolic burden, as measured by traditional risk factors and CVD history. Validation with LCA confirmed the robustness of the classification, and disease-stratified analyses in PsO and PsA further supported the reproducibility of the phenotypes across disease subtypes.

Our findings both relate to and expand upon previous evidence published by our group, where a classical analysis of three cohorts using regression and meta-analysis revealed an inverse association between HLA-Cw6 and diabetes, particularly in patients with psoriasis without arthritis, supporting the existence of an HLA-Cw6-linked cardiometabolic “protective” endotype [9]. However, that work did not assess within-disease heterogeneity or explore potential differences between psoriasis and psoriatic arthritis beyond multivariable adjustments. By incorporating HLA-Cw6 as a variable in an unsupervised clustering framework, the present study partially confirms that protective pattern in PsA, yet uncovers an opposite trend in PsO, where Cw6 positivity was associated with a high-cardiometabolic-risk phenotype. This observation suggests that the impact of HLA-Cw6 on cardiometabolic risk may be context-dependent, modulated by the predominant clinical domain (cutaneous versus articular) and other factors such as age and disease duration. Therefore, combining classical association analyses with integrative phenotyping techniques provides a more comprehensive view of the complex interplay between genetics, clinical expression, and comorbidity across the psoriatic disease spectrum.

The multivariable models consistently demonstrated that age and cardiometabolic burden, rather than disease-specific or genetic factors, were the main determinants of the high-risk cluster in the whole population. This finding was robust across different model specifications—comprehensive, clinically adjusted, and parsimonious—underscoring the stability of the association between traditional cardiovascular risk factors and the systemic expression of psoriatic disease. The absence of significant effects for PASI, PsA status, or HLA-Cw6 after adjustment suggests that the transition toward a cardiometabolic-dominant phenotype is more closely linked to aging and cumulative metabolic stress than to inflammatory or genetic drivers. BMI differences across clusters were modest. This reflects two offsetting phenomena: (i) the smoking-heavy, younger psoriasis clusters displayed lower BMI, and (ii) older, comorbidity-burdened individuals in the high-risk cluster may have reduced weight relative to earlier life. These opposing trends likely attenuate BMI separation across clusters. Overall, these results reinforce the concept that cardiometabolic comorbidity in psoriatic disease is not merely an epiphenomenon but an integral component of disease heterogeneity, with implications for risk stratification and tailored preventive strategies.

That said, the inclusion of HLA-Cw6—a validated immunogenetic marker—in our clustering variables adds a biological dimension to the identified groups. This supports the interpretation of these subgroups as candidate endotypes: data-driven phenotypes potentially underpinned by distinct pathogenic mechanisms. Previous studies have linked HLA-Cw6 to type I psoriasis, characterized by early onset, extensive skin disease, strong family history, and lower prevalence of obesity and metabolic syndrome compared with Cw6-negative psoriasis [10,11,12]. Our stratified analysis revealed a divergent pattern in the relationship between HLA-Cw6 and cardiometabolic risk across the PsD spectrum. Among patients with PsO only, HLA-Cw6 positivity was associated with the high-risk cardiometabolic cluster. Conversely, in PsA, Cw6 positivity was substantially less frequent in the high-risk cluster and more common in the lower-risk phenotypes. By incorporating HLA-Cw6 into the unsupervised clustering, we partly confirm the previously described protective cardiometabolic pattern in PsA, yet we also uncover an opposite association in psoriasis-only patients. This suggests that the impact of HLA-Cw6 on cardiometabolic burden may be context-dependent, influenced by the predominant clinical domain (cutaneous versus articular), age, and disease duration. This phenotypic inversion between PsO and PsA underscores the potential role of HLA-Cw6 not only as a marker of cutaneous disease susceptibility but also as a potential stratifier of cardiometabolic risk within the broader PsD spectrum [13,14,15]. The findings align with the growing recognition that genetic markers, when integrated with clinical and metabolic profiles, can refine endotype definitions and guide risk-based management strategies [13,14,15]. However, validation of our results as true pathobiologically distinct endotypes will require longitudinal designs and multiomics integration (e.g., cytokines, transcriptomics, and metabolomics).

The principal component analysis (PCA) provided an integrative visualization of the multidimensional structure underlying psoriatic disease. The first two components accounted for most of the between-cluster variance and reflected two major and biologically meaningful axes: a cardiometabolic dimension (PC1) and an inflammatory/immunogenetic dimension (PC2). Variables such as hypertension, diabetes, dyslipidemia, and cardiovascular disease loaded strongly on PC1, whereas PsA, female sex, and systemic therapy contributed primarily to PC2. The inverse loading of HLA-Cw6 on PC1 further supports the dissociation between genetic susceptibility and metabolic burden. The negative loading of HLA-Cw6 on the cardiometabolic axis is biologically plausible, reflecting its established association with type I psoriasis, earlier onset, and a comparatively lower prevalence of obesity and metabolic syndrome [10,11]. Together, these findings confirm that cardiometabolic risk factors and inflammatory pathways cluster along partially independent axes yet converge in patients with more severe systemic disease (cluster C4). This pattern highlights the coexistence of metabolic and immune-driven mechanisms in psoriatic disease, consistent with prior multidimensional models linking low-grade inflammation, adiposity, and psoriatic manifestations [13,14,15,16,17].

Methodologically, the classification showed high agreement between k-means and LCA (adjusted Rand index 0.83), supporting reproducibility. The bootstrap procedure in regression analysis ensured stable estimates despite the small size of high-risk clusters. The disease-stratified analyses further confirmed that the high-risk phenotype is not confined to those with arthritis.

Our results have several implications. First, a comprehensive CV risk assessment in all PsD—High-risk phenotypes were present in both PsO and PsA, challenging the assumption that arthritis alone drives systemic risk [16,17]. Second, potential genetic stratification—Cw6 status could help identify patients more likely to present with lower metabolic burden, particularly in PsA, whereas Cw6-negative status may alert clinicians to higher CV risk. Third, targeted prevention—small, high-risk clusters such as C4, PsO-S3, and PsA-S3 represent priority groups for aggressive CV risk factor control and possibly closer dermatology–rheumatology–cardiology collaboration. Fourth, lifestyle interventions—phenotypes characterized by high smoking prevalence (C3, PsO-S2) highlight modifiable risks that may be overlooked in PsD management [18]. Fifth, research implications—these clusters provide a framework for studying differences in therapeutic response and long-term outcomes across phenotypes. From a clinical perspective, our findings caution against assuming that PsA uniformly carries greater cardiometabolic risk than PsO alone. Instead, both entities contain small, high-risk subgroups characterized by older age, established CVD, and clustering of traditional risk factors. The opposite HLA-Cw6 patterns in the psoriasis-only versus PsA high-risk phenotypes further support the notion of disease-specific cardiometabolic trajectories within the broader psoriatic spectrum.

Of course we must highlight some drawbacks. The cross-sectional design limits causal inference. No independent external cohort was available, and this is a limitation. Systemic therapy was included as a proxy of disease severity, but treatment duration and response were not available. Therefore, we cannot exclude residual confounding related to treatment history. While the inclusion of HLA-Cw6 provides a biological anchor, we lacked other biomarkers (e.g., inflammatory cytokines, metabolomics) that could deepen mechanistic understanding. In the PsA-stratified analysis, the smallest high-cardiometabolic-risk subgroup (PsA-S3, n = 14) should be interpreted as a candidate phenotype rather than a definitive endotype, given its limited size and the potential sensitivity to sampling variability. Additionally, clustering was based on variables available in clinical practice; adding longitudinal trajectories may refine phenotyping. Given the cross-sectional design, our analysis can only describe patterns of co-occurrence, not causal pathways or temporal trajectories. Therefore, any reference to potential long-term cardiometabolic courses should be interpreted as hypothesis-generating.

Longitudinal studies should test the stability of these phenotypes and their predictive value for CV events, treatment response, and disease progression. Integrating multi-omics data could confirm biological distinctiveness, transitioning from candidate endotypes to validated endotypes. Interventional trials targeting high-risk groups could determine whether phenotype-guided prevention improves outcomes [19,20].

## 5. Conclusions

In this study of 572 patients with psoriatic disease, we identified four distinct cardiometabolic phenotypes using unsupervised clustering. The results were robust to model-based validation and consistent within psoriasis-only and psoriatic arthritis strata. The high-risk phenotype was largely driven by older age and cumulative cardiometabolic burden, while the use of bootstrap resampling provided stable confidence intervals, reinforcing the reliability of these associations despite the small size of this subgroup. Our findings suggest that simple demographic and clinical variables can stratify PsD patients into reproducible cardiometabolic endotypes with distinct preventive and therapeutic needs. Integrating such phenotyping into routine clinical practice could help tailor cardiovascular risk assessment and guide targeted interventions aimed at improving long-term outcomes.

## Figures and Tables

**Table 1:** Disease characteristics of the study cohort and sex-based differences.

Variables	Total n: 572	Men n: 309	Women n: 263
Age (yrs), mean (SD)	46.7 (14.5)	47.5 (14.6)	45.7 (14.3)
Age at disease onset (yrs), median (min, max)	23.0 [1.0, 78.0]	24.5 [1.0, 74.0]	20.0 [1.0, 78.0]
Education level:			
Primary, n (%)	163 (28.5)	111 (35.9)	52 (19.8)
Secondary, n (%)	262 (45.8)	130 (42.1)	132 (50.2)
University, n (%)	147 (25.7)	68 (22.0)	79 (30.0)
Duration (yrs), mean (SD)	19.4 (14.8)	18.7 (14.3)	20.3 (15.4)
PsA, n (%)	171 (30)	81 (26.2)	90 (34.2)
Weight, mean (SD)	77.9 (16.4)	85.4 (14.6)	69.3 (13.9)
BMI, mean (SD)	27.6 (5.02)	28.5 (4.43)	26.7 (5.48)
Waist perimeter (cm), mean (SD)	96.7 (14.1)	101 (11.6)	91.4 (14.7)
PASI, mean (SD)	15.1 (12.0)	16.0 (11.9)	14.0 (12.0)
PASI ≥ 10, n (%)	294 (51.4%)	174 (56.3)	120 (45.6%)
Nail disease, n (%)	328 (57.3%)	192 (62.1)	136 (51.7%)
Plaque psoriasis, n (%)	497 (86.9)	281 (90.9)	216 (82.1)
Smoking, n (%)	196 (34.3)	99 (32.0)	97 (36.9)
Alcohol consumption (SDU), median (min, max)	0 [0.0, 30.0]	0 [0.0, 2.0]	0 [0.0, 30.0]
T1D, n (%)	22 (3.8)	11 (3.6)	11 (4.2)
T2D, n (%)	45 (7.9)	29 (9.4)	16 (6.1)
Hypertension, n (%)	114 (20.0)	68 (22.0)	46 (17.5)
Dyslipidemia, n (%)	113 (19.8)	68 (22.0)	45 (17.1)
NAFLD, n (%)	129 (22.6)	99 (32.0)	30 (11.4)
Patients with CVD, n (%)	33 (5.8)	20 (6.5)	13 (4.9)
Patients on systemic therapy, n (%)	370 (64.7)	203 (65.7)	167 (63.5)
Biologics, n (%)	233 (40.7)	125 (40.5)	108 (41.1)
Anti-TNF	118 (20.6)	63 (20.4)	55 (20.9)
Anti IL12/23	63 (11.0)	35 (11.3)	28 (10.6)
Anti-IL17	52 (9.1)	27 (8.8)	25 (9.5)

yrs: years; PsA: psoriatic arthritis; BMI: body mass index; PASI: psoriasis area and severity index; SDU: standard drink unit; T1D: type 1 diabetes; T2D: type 2 diabetes; NAFLD: non-alcoholic fatty liver disease; CVD: cardiovascular disease; TNF: tumor necrosis factor; IL: interleukin.

**Table 2:** Overall cluster summary (k-means, k = 4).

Characteristics	Cluster 1 n: 217	Cluster 2 n: 144	Cluster 3 n: 178	Cluster 4 n: 33
Age, years, median (IQR)	45(34–57)	49 (39.8–56.2)	43.5 (33.2–53.0)	66 (60–72)
Disease duration, years, median (IQR)	17(8–30)	23 (16–34)	9 (3–20)	30(10–49)
Women (%)	41	53.5	47.2	39.4
PsA (%)	0	100	7.3	42.4
HLA-Cw6 (%)	49.3	39.6	37.6	30.3
PASI, median (IQR)	12(9–20)	12 (8–20)	4 (3–6)	7 (3–14)
Systemic therapy (%)	99.5	93.8	0	57.6
CVD (%)	0	0	0	100
Hypertension (%)	13.8	20.8	16.3	75.8
Diabetes (%)	7.4	13.2	9.6	45.5
Dyslipidemia (%)	13.4	25	14	69.7
Obesity (%)	26.3	28.5	29.8	30.3
Smoking (%)	35.5	28.5	41	15.2
NAFLD (%)	22.7	25.4	23.6	27.3
Number of CVRF, median (IQR)	1 (0–2)	1 (0.8–2)	1 (0.2–2)	3 (2–4)

IQR: interquartile range; PsA: psoriatic arthritis; HLA: human leukocyte antigen; PASI: psoriasis area and severity index; CVD: cardiovascular disease; NAFLD: non-alcoholic fatty liver disease; CVRF: cardiovascular risk factors. Consistent with the binary dyslipidemia variable, patients in the high-risk cluster displayed a more atherogenic lipid profile compared with the remaining clusters (Table S2).

**Table 3:** Psoriasis-only clusters (k = 3).

Characteristics	Cluster 1, n: 217	Cluster 2, n: 165	Cluster 3, n: 19
Age, years, median (IQR)	45 (34–57)	44 (33–53)	66 (58.5–69.5)
Disease duration, years, median (IQR)	17 (8–30)	9 (3–21.2)	21 (8–46.5)
Women (%)	41	47.3	31.6
HLA-Cw6 (%)	34.6	48.5	57.9
PASI, median (IQR)	12 (9–20)	4 (3–6)	8 (3.2–13.5)
Systemic therapy (%)	99.5	0	68.4
CVD (%)	0	0	100
Hypertension (%)	13.8	15.2	68.4
Diabetes (%)	7.4	9.7	42.1
Dyslipidemia (%)	13.4	13.9	68.4
Obesity (%)	26.3	29.7	47.4
Smoking (%)	35.5	40.6	15.8
NAFLD (%)	21.7	23	15.8
Number of CVRF, median (IQR)	1 (0–2)	1 (1–2)	2 (2–4)

IQR: interquartile range; HLA: human leukocyte antigen; PASI: psoriasis area and severity index; CVD: cardiovascular disease; NAFLD: non-alcoholic fatty liver disease; CVRF: cardiovascular risk factors.

**Table 4:** Psoriatic arthritis clusters (k = 3).

Characteristics	Cluster 1, n: 93	Cluster 2, n: 64	Cluster 3, n: 14
Age, years, median (IQR)	52 (39–59)	47 (39.8–53)	67 (60.5–75)
Disease duration, years, median (IQR)	19 (12–27)	26 (16–34.2)	37 (10.2–51.5)
Women (%)	47.3	60.9	50
HLA-Cw6 (%)	46.2	48.4	21.4
PASI, median (IQR)	12 (7.4–20)	12 (6.1–16)	4 (3–15)
Systemic therapy (%)	89.2	81.2	42.9
CVD (%)	0	0	100
Hypertension (%)	25.8	15.6	85.7
Diabetes (%)	16.1	7.8	50
Dyslipidemia (%)	23.7	25	71.4
Obesity (%)	33.3	21.9	7.1
Smoking (%)	26.9	34.4	14.3
NAFLD (%)	21.5	23.4	42.9
Number of CVRF, median (IQR)	1 (1–2)	1 (0–2)	3 (2–3.8)

IQR: interquartile range; HLA: human leukocyte antigen; PASI: psoriasis area and severity index; CVD: cardiovascular disease; NAFLD: non-alcoholic fatty liver disease; CVRF: cardiovascular risk factors.

**Table 5:** Contribution of each variable to principal component analysis.

Variable	PC1	PC2	Interpretation
Hypertension (%)	+0.37	−0.09	Strong positive contribution to PC1 (cardiometabolic axis)
Diabetes (%)	+0.37	−0.07	Same direction as hypertension and dyslipidemia
Dyslipidemia (%)	+0.36	−0.16	Same direction as hypertension and diabetes
Cardiovascular disease (%)	+0.36	−0.16	Strong weight in PC1, aligned with age and higher risk
Age/Disease duration	≈0	≈0	No differential contribution (already captured in cardiovascular disease)
HLA-Cw6 (%)	−0.33	−0.05	Negative direction in PC1 → associated with low cardiometabolic risk
Systemic therapy (%)	−0.04	+0.31	Reflects (proxy) inflammatory activity, more clearly in PC2
PsA (%)	+0.11	+0.67	Strongest contributor to PC2
Women (%)	−0.14	+0.58	Strong contributor to PC2

Loadings indicate the contribution and direction (positive or negative) of each variable to the respective component. Values > |0.3| were considered relevant for interpretation.

## Data Availability

Data supporting the findings of this study are available from the corresponding author upon reasonable request. Due to patient confidentiality and institutional policies, raw individual-level data cannot be made publicly available.

## Supplementary Materials

The following supporting information can be downloaded at: https://www.mdpi.com/article/10.3390/life16010002/s1. STROBE Reporting Statement: This manuscript was prepared and reported in accordance with the STROBE guidelines for observational studies (see Supplementary Material). Table S1: STROBE Checklist–Cardiometabolic Endotypes in Psoriatic Disease (Cross-Sectional Study); Table S2: Lipid profile across clusters; Table S3: Bootstrap-derived 95% confidence intervals for key cardiometabolic variables in the high-risk cluster (C4); Table S4: Interaction model (HLA-Cw6 × PsA); Table S5: Multivariable Analysis: High-risk cluster (C4) vs others; Table S6: Collinearity diagnostics (VIF)–Model 1 predictors; Figure S1: Correlation matrix of clustering variables; Figure S2: Sensitivity clustering including BMI and waist circumference; Figure S3: PCA excluding age (age-insensitive structure).
