# Supplementary material for: Cardiometabolic Candidate Endotypes in Psoriatic Disease: Integration of Clinical, Metabolic, and Immunogenetic Data Across Psoriasis and Psoriatic Arthritis
Source: Life (Basel). 2025 Dec 19;16(1):2. doi: 10.3390/life16010002 (PMC12843029; doi:10.3390/life16010002)
Supplement: Supplementary file 1 [file life-16-00002-s001.zip › life-4019771-supplementary.pdf]

Supplementary material

Table S1. STROBE Checklist – Cardiometabolic Endotypes in Psoriatic Disease (Cross-Sectional Study)

Item No.	Recommendation (STROBE)	Section/Paragraph where addressed	Notes
1	Indicate the study's design with a commonly used term in the title or the abstract	Title; Abstract	Fully addressed
2	Explain the scientific background and rationale for the investigation	Introduction – Background	Fully addressed
3	State specific objectives, including any prespecified hypotheses	Introduction – Objectives	Fully addressed
4	Present key elements of study design early in the paper	Methods – Study design and population	Fully addressed
5	Describe the setting, locations, and relevant dates, including periods of recruitment and data collection	Methods – Study design and population	Fully addressed
6	Give the eligibility criteria, and the sources and methods of selection of participants	Methods – Study design and population	Fully addressed
7	Clearly define all outcomes, exposures, predictors, potential confounders, and effect modifiers	Methods – Variables	Fully addressed
8	For each variable of interest, give sources of data and details of	Methods – Variables; Data preprocessing	Fully addressed

	methods of assessment		
9	Describe any efforts to address potential sources of bias	Discussion – Limitations	Partially addressed (selection bias mentioned)
10	Explain how the study size was arrived at	Methods – Study design and population	Partially addressed (consecutive sampling)
11	Explain how quantitative variables were handled in the analyses	Methods – Data preprocessing	Fully addressed
12	Describe all statistical methods, including those used to control for confounding	Methods – Clustering procedures; Validation and additional analyses	Fully addressed
13	Describe participants (numbers, characteristics, etc.)	Results – Summary of study population; Table 1	Fully addressed
14	Give numbers of participants with missing data for each variable of interest	Methods – Data preprocessing	Fully addressed (imputation described)
15	Report descriptive data for each variable of interest	Results – Summary of study population	Fully addressed
16	Report main results (unadjusted and adjusted estimates, precision, etc.)	Results – Predictors of high-risk phenotype	Fully addressed
17	Other analyses done—e.g., subgroups, interactions, sensitivity analyses	Results – Disease-stratified clustering; Validation with Gaussian Mixture Models	Fully addressed
18	Summarise key results with reference to study objectives	Discussion – first paragraph	Fully addressed
19	Discuss limitations of the study, taking into account potential sources of bias or imprecision	Discussion – Limitations	Fully addressed
20	Give a cautious overall interpretation considering objectives,	Discussion – Final paragraphs	Fully addressed

	limitations, and other evidence		
21	Discuss the generalisability (external validity) of the study results	Discussion – Limitations	Partially addressed (single-centre)
22	Give the source of funding and the role of the funders	Declarations – Funding	Fully addressed

Table S2. Lipid profile across clusters.

Lipid Parameter	C1	C2	C3	C4 (High-risk)
**Total Cholesterol**	204.29 ± 42.44	203.44 ± 41.94	204.13 ± 38.71	**189.42 ± 45.07**
**LDL**	127.23 ± 37.36	126.70 ± 35.72	128.19 ± 33.88	**115.97 ± 38.07**
**HDL**	55.10 ± 17.12	53.30 ± 14.13	52.36 ± 14.51	**47.21 ± 10.39**
**Triglycerides**	114.86 ± 65.66	129.35 ± 77.10	127.75 ± 81.13	**151.55 ± 91.07**

Lipid profile characteristics by cluster. Values shown as mean ± standard deviation. The high-risk cluster (C4) shows the most atherogenic profile, with lower HDL, higher triglycerides, and lower LDL levels in the context of universal CVD and heavy metabolic burden, consistent with advanced cardiometabolic disease. The remaining clusters show comparable lipid values with modest variations. Although the high-risk cluster (C4) exhibited the lowest LDL levels, this is consistent with universal CVD and likely widespread use of lipid-lowering therapy. The more atherogenic pattern (higher triglycerides and lower HDL) aligns with advanced cardiometabolic disease despite pharmacologically controlled LDL.

Table S3. Bootstrap-derived 95% confidence intervals for key cardiometabolic variables in the high-risk cluster (C4)

Variable	Mean Estimate	95% CI (Lower)	95% CI (Upper)
Hypertension	0.76	0.61	0.88
Diabetes	0.45	0.30	0.64
Dyslipidemia	0.70	0.55	0.85
NAFLD	0.27	0.12	0.42
Smoking	0.15	0.03	0.27
BMI (kg/m^2)	28.05	26.32	29.83

(B = 2000 resamples). Table S3 presents bootstrap-based internal validation of the C4 high-risk phenotype. Confidence intervals remained narrow for all variables, confirming the stability of C4 despite its modest size (n = 33).

Table S4. Interaction model (HLA-Cw6 × PsA)

**Predictor**	**β (Coefficient)**	**SE**	**z**	**p-value**	**95% CI**
Intercept	–3.07	0.32	–9.48	<0.001	–3.70 to –2.43
HLA-Cw6	0.15	0.47	0.31	0.754	–0.78 to 1.07
PsA	1.10	0.44	2.51	0.012	0.24 to 1.96
Cw6 × PsA	–2.34	1.15	–2.03	0.042	–4.59 to –0.08

The significant interaction ($p = 0.042$) demonstrates disease-specific immunogenetic effects, with HLA-Cw6 associated with higher cardiometabolic risk in PsO but lower risk in PsA.

Table S5. Multivariable Analysis: High-risk cluster (C4) vs others

Model 1 – Full

Variable	OR	95% CI (low)	95% CI (high)	p-value
Age	1.08	1.03	1.12	0.0012
Sex	0.93	0.38	2.29	0.8813
PsA	0.94	0.36	2.45	0.9022
PASI	0.99	0.94	1.03	0.6044
Cw6	1.0	0.38	2.62	0.9996
Systemic therapy	0.71	0.26	1.92	0.4976
Hypertension	3.7	1.3	10.58	0.0145
Diabetes	1.68	1.02	2.76	0.0403
Dyslipidemia	4.5	1.76	11.51	0.0017
Obesity	0.41	0.15	1.1	0.0758

Model 2 – Clinically adjusted (univariate p<0.10)

Variable	OR	95% CI (low)	95% CI (high)	p-value
Age	1.08	1.03	1.12	0.0007
Hypertension	3.0	1.13	7.96	0.0273
Diabetes	1.59	0.98	2.56	0.0586
Dyslipidemia	4.3	1.74	10.62	0.0015

Model 3 – Backward stepwise

Variable	OR	95% CI (low)	95% CI (high)	p-value
Age	1.07	1.03	1.12	0.0015
Hypertension	3.66	1.32	10.1	0.0124

Diabetes	1.69	1.03	2.75	0.0366
Dyslipidemia	4.65	1.86	11.64	0.001
Obesity	0.45	0.17	1.16	0.0987

Table S6. Collinearity diagnostics (VIF) – Model 1 predictors

Variable	VIF
Age	1.42
Sex	1.03
PsA	1.11
PASI	1.26
Cw6	1.06
Systemic therapy	1.35
Hypertension	1.41
Diabetes	1.21
Dyslipidemia	1.22
Obesity	1.11

Figure S1 – Correlation matrix of clustering variables

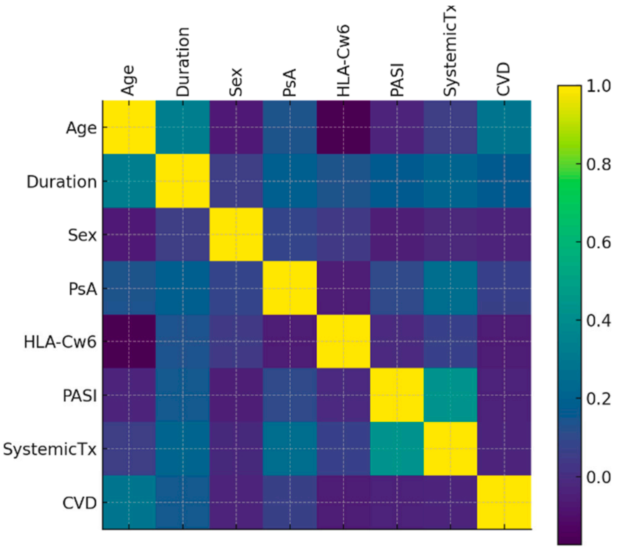

Correlation matrix of the eight clustering variables (age, disease duration, sex, PsA status, HLA-Cw6, PASI, systemic therapy, CVD). All pairwise correlations are moderate (|r| ≤ ~0.4), supporting their joint use in k-means clustering without evidence of problematic multicollinearity.

Figure S2 – Sensitivity clustering including BMI and waist circumference

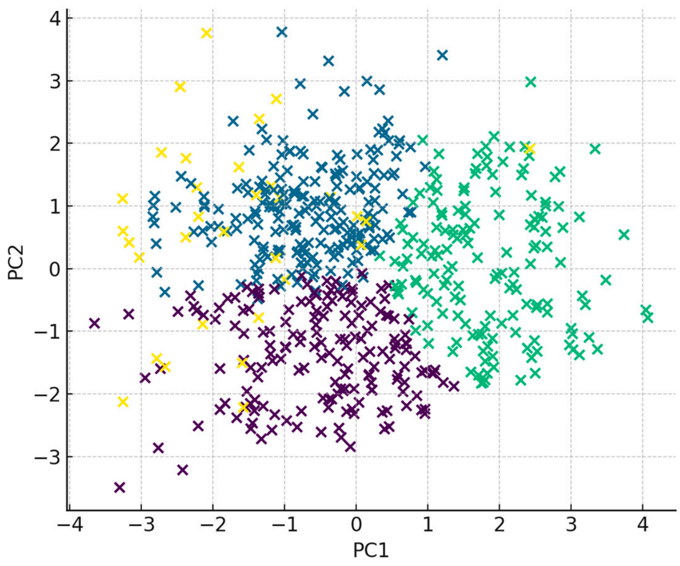

Sensitivity k-means clustering including BMI and waist circumference. A principal component analysis (PCA) projection of the extended variable space (age, disease duration, sex, PsA status, HLA-Cw6, PASI, systemic therapy, CVD, BMI, waist circumference) is shown, with points colored according to the 4-cluster solution. The small high-risk phenotype remains identical in membership (100% overlap) compared with the primary clustering model, confirming its robustness to inclusion of anthropometric variables.

Figure S3 – PCA excluding age (age-insensitive structure)

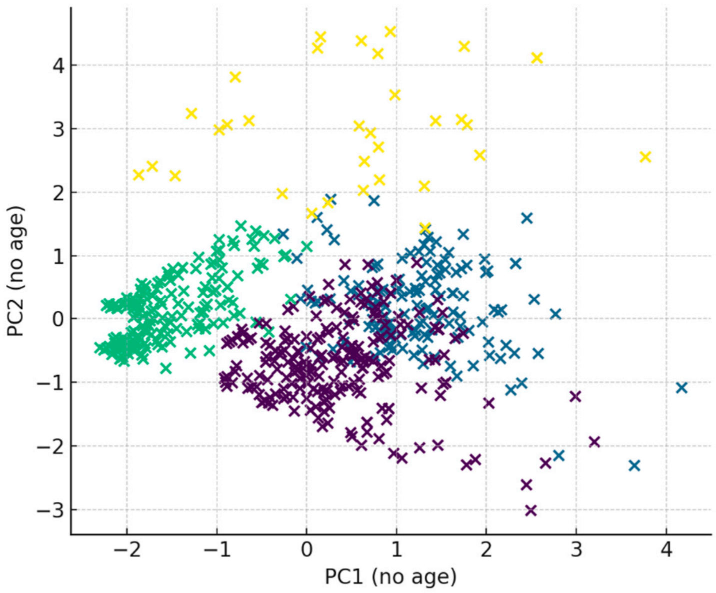

Principal component analysis (PCA) of the clinical–cardiometabolic variable set excluding age (Duration, sex, PsA status, HLA-Cw6, PASI, systemic therapy, CVD). The scatterplot depicts PC1 vs PC2, with points colored by the original 4-cluster solution. The same cardiometabolic axis and cluster separation are preserved even when age is omitted from the model, indicating that the clustering structure is not driven solely by chronological age.
